# Supplementary material for: Oxidation of Microcystis aeruginosa and Microcystins with Peracetic Acid
Source: Toxins (Basel). 2024 Jul 23;16(8):328. doi: 10.3390/toxins16080328 (PMC11360697; doi:10.3390/toxins16080328)
Supplement: Supplementary file 1 [file toxins-16-00328-s001.zip › toxins-3088841-supplementary.pdf]

Supplementary Materials: Oxidation of *Microcystis aeruginosa* and Microcystins with Peracetic Acid

Mennatallah Alnahas, Husein Almuhtaram and Ron Hofmann

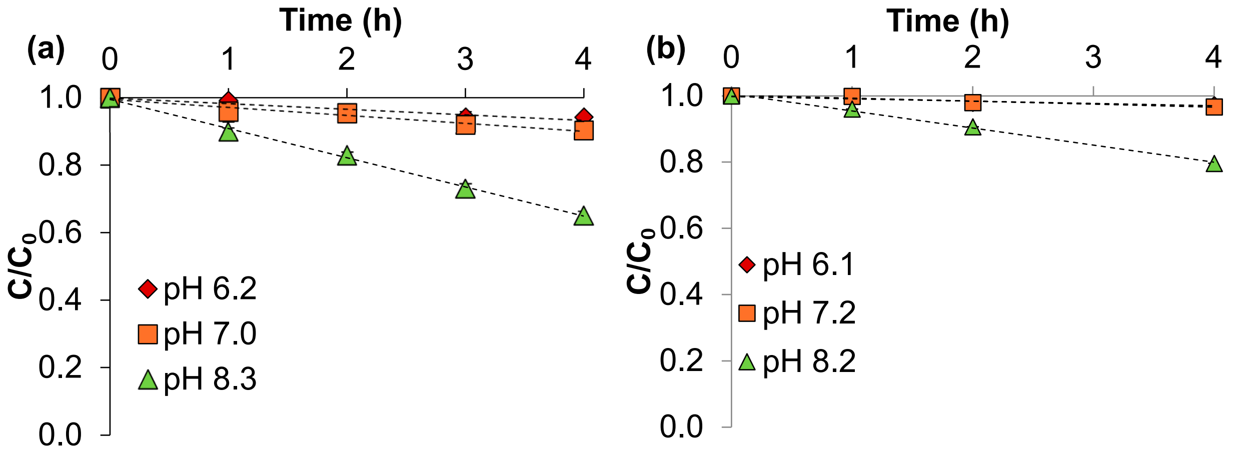

Figure S1. Degradation of 50 µg/L of **a**) MC-LR and **b**) -RR in Milli-Q water using 10 mg/L of PAA. Error bars represent duplicate trials

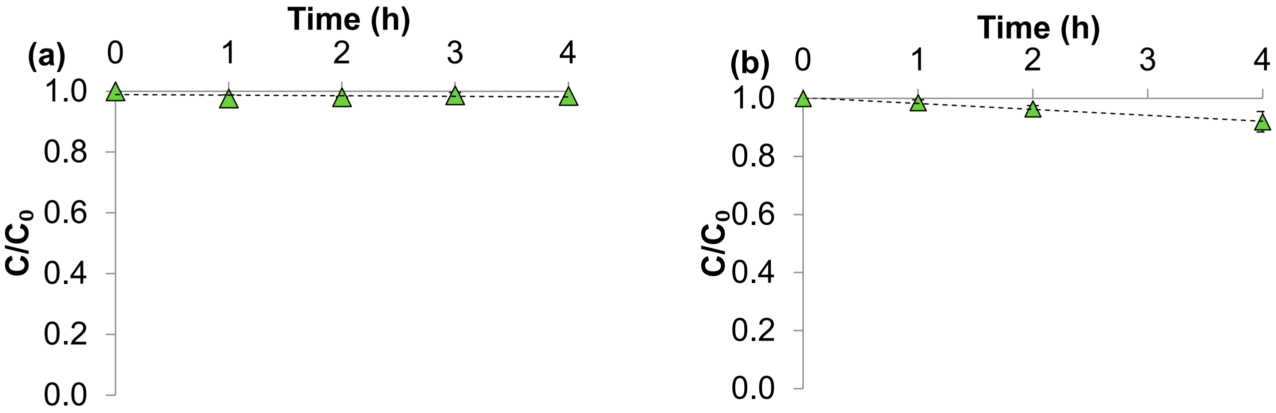

Figure S2. Degradation of 50 µg/L of **a**) MC-LR and **b**) -RR in Milli-Q water using 1.5 mg/L of H_2O_2. Error bars represent duplicate trials.

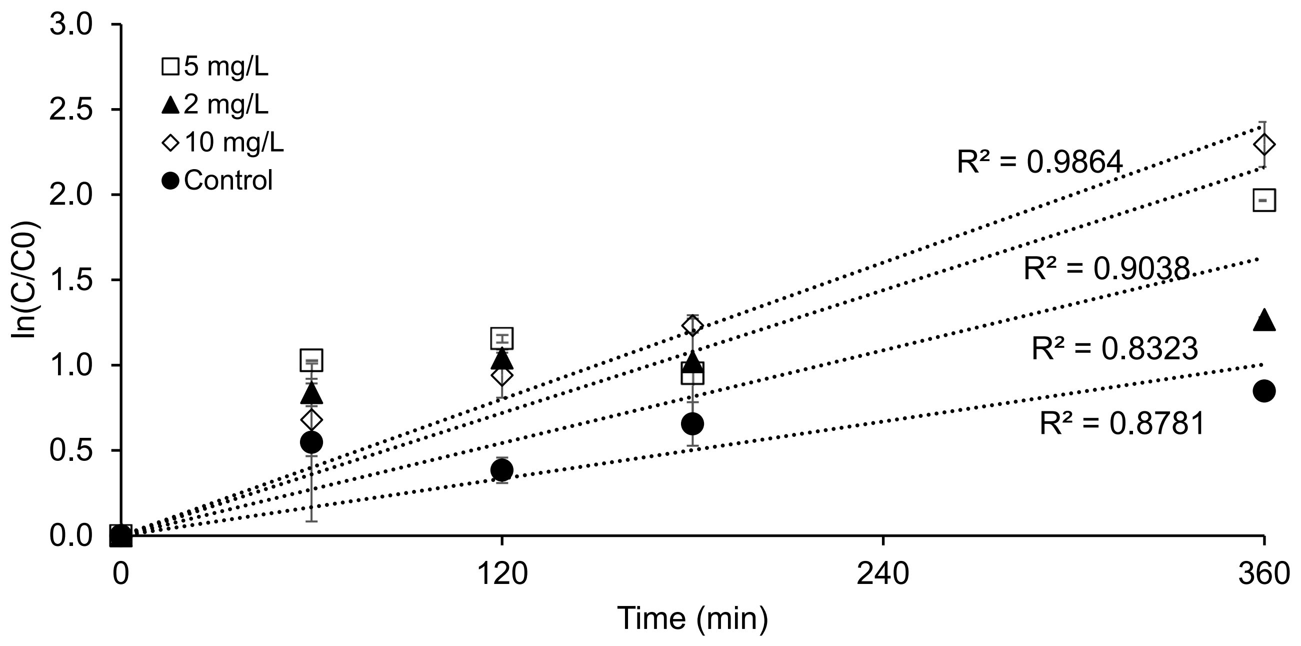

Figure S3. Release of cell-bound MC-LR with different PAA doses from 1 × 10^6 cell/ml *M. aeruginosa*. Error bars represent standard deviations of triplicate trials.

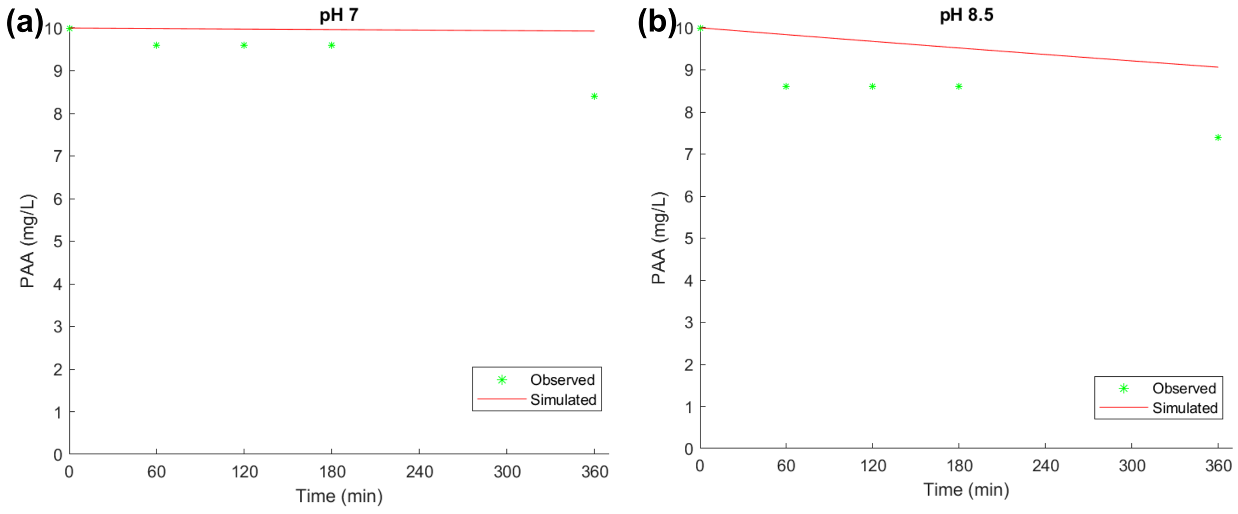

Figure S4. Observed PAA decay in the presence of 1 × 10^6 *M. aeruginosa* versus simulated PAA decay in Milli-Q water adopted from Chen et al [6] at **a**) pH 7 and **b**) pH 8.5 using 10 mM phosphate buffer.
